# Long-Term Follow-Up, Association between CARD15/NOD2 Polymorphisms, and Clinical Disease Behavior in Crohn's Disease Surgical Patients

**DOI:** 10.1155/2021/8854916

**Published:** 2021-02-24

**Authors:** Francesco Giudici, Tiziana Cavalli, Cristina Luceri, Edda Russo, Daniela Zambonin, Stefano Scaringi, Ferdinando Ficari, Marilena Fazi, Amedeo Amedei, Francesco Tonelli, Cecilia Malentacchi

**Affiliations:** ^1^Department of Clinical and Experimental Medicine, Surgical Unit, University of Florence, Italy; ^2^Department of Neuroscience, Psychology, Pharmacology and Child Health (NEUROFARBA), Italy; ^3^Department of Biomedical Experimental and Clinical Sciences, “Mario Serio”, Italy

## Abstract

**Background:**

CARD15/NOD2 is the most significant genetic susceptibility in Crohn's disease (CD) even though a relationship between the different polymorphisms and clinical phenotype has not been described yet. The study is aimed at analyzing, in a group of CD patients undergoing surgery, the relationship between CARD15/NOD2 polymorphisms and the clinical CD behavior after a long-term follow-up, in order to identify potential clinical biomarkers of prognosis.

**Methods:**

191 surgical CD patients were prospectively characterized both for the main single nucleotide polymorphisms of CARD15/NOD2 and for many other environmental risk factors connected with the severe disease form. After a mean follow-up of 7.3 years, the correlations between clinical features and CD natural history were analyzed.

**Results:**

CARD15/NOD2 polymorphisms were significantly associated with younger age at diagnosis compared to wild type cases (*p* < 0.05). Moreover, patients carrying a 3020insC polymorphism presented a larger Δ between diagnosis and surgery (*p* = 0.0344). Patients carrying an hz881 and a 3020insC exhibited, respectively, a lower rate of responsiveness to azathioprine (*p* = 0.012), but no difference was found in biologic therapy. Finally, the risk of surgical recurrence was significantly associated, respectively, to age at diagnosis, to familial CD history, to diagnostic delay, to arthritis, and to the presence of perioperative complications.

**Conclusions:**

3020insC CARD15 polymorphism is associated with an earlier CD onset, and age at CD diagnosis < 27 years was confirmed to have a detrimental effect on its clinical course. In addition, the familiarity seems to be connected with a more aggressive postoperative course. Finally, for the first time, we have observed a lower rate of responsiveness to azathioprine in patients carrying an hz881 and a 3020insC.

## 1. Introduction

Crohn's disease (CD (MIM 266600)) is classified as an idiopathic inflammatory bowel disease (IBD) with a multifactorial pathogenesis [1–3]. The role of genetics in the development of CD began to be outlined in the 1980s, based on studies showing an increased number of cases among patients' family members [4]. Genome-wide association studies (GWAS) provided the identification of up to 163 loci in the human genome associated with IBD, significantly more than that in any other complex diseases [5, 6–13].

In addition, several risk factors for CD development have been reported, but it is still unknown if their pathogenic mechanism could be mediated by genetics: smoking habit is probably the most studied one [14, 15–23], and medical and surgical recurrence rates are significantly higher in smoking CD patients [24–28, 29, 30]. Previous appendectomy seems another risk factor [31–33, 34, 35]; also, the use of antibiotics during the first years of life seems to be associated with early CD development [36–38], and similar experiences in adults who underwent long-lasting prior antibiotic or NSAID use are reported too [39–44]; breastfeeding seems protective for CD [45–48].

Little experiences reported that patients carrying NOD2/CARD15 polymorphisms could develop a more aggressive form of Crohn's disease showing a trend for an early surgery followed by multiple surgical interventions [13]. However, a real clinical definition of aggressiveness is still lacking, and the role of genetic factors in the clinical CD history is still undefined.

In this monocentric study, we have enrolled a cohort of patients characterized by a severe CD disease requiring surgery and analyzed the relationship between the different polymorphisms and the postoperative clinical disease behavior after a long-term follow-up in order to detect potential biomarkers of Crohn's disease course.

## 2. Materials and Methods

### 2.1. Patients

After Ethical Committee approval and informed consent signature, prospectively, peripheral blood samples were collected from 191 Caucasian CD patients consecutively admitted for abdominal surgical operation to the IBD surgical unit of the Department of Surgery and Translational Medicine of the University of Florence, during the period January 2010-December 2014. No other exclusion criteria were adopted in our prospective observational study, characterized by a mean follow-up of 7.3 years (range 5.1-9.2). Examined clinical data included gender, age at diagnosis, family history of IBD, presence of extraintestinal or perianal disease, number of IBD-associated surgeries and intersurgical interval, recurrence of disease, surgical complications, and status of disease. Demographic and clinical features of patients are reported in Table 1.

### 2.2. Genomic DNA Extraction

Genomic DNA of patients was extracted from peripheral blood leukocytes with a Wizard Genomic DNA Purification Kit (Promega, Madison, WI, USA) according to the manufacturer's instructions and quantified by a NanoDrop-1000 Spectrophotometer (NanoDrop Technologies, Wilmington, DE, USA).

### 2.3. CARD15/NOD2 Gene Polymorphism Analysis

**Figure 1:**
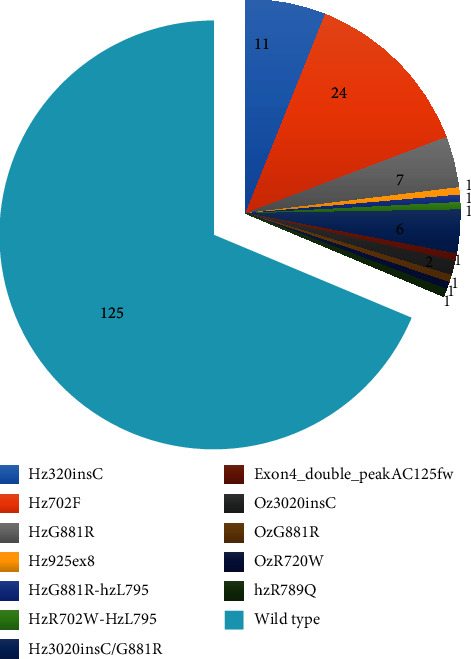
Genotype distribution of the clinical sample population. The figure shows the distribution of *CARD15/NOD2* genotypes detected in the examined cohort of surgical patients (number of patients).

Exons 4, 8, and 11 of the NOD2 gene were amplified by PCR for the detection of R702W, G881R, and 3020insC polymorphisms (representing 32%, 18%, and 31%, respectively, of the total CD polymorphisms). They were sequenced using specific couples of primers and PCR condition as reported in Table 2. The distribution of detected genotypes of our surgical patients is shown in Figure 1 and Table 3.

R702W and 3020insC polymorphism were screened by PCR-based sequencing reaction, using a BigDye Terminator Purification Kit (Applied Biosystems, Foster City, CA, USA) and the ABI Prism 310 Genetic Analyzer (Applied Biosystems). G881R polymorphism has been screened by PCR-based enzymatic digestion using HhaI restriction endonuclease (Fermentas, Burlington, Canada).

Through sequencing reaction and microcapillary electrophoresis in a ABI Prism 310 automated sequencer (Applied Biosystems®, Foster City, Calif., USA), electropherogram by fluorescence detection was obtained. Clinical and anamnestic data collected for the CD patients—including age of disease onset, disease location and severity, and smoking habit—were analyzed and correlated with the genetic profile.

### 2.4. Statistical Analysis

Statistical analyses were performed using the STATGRAPHICS Centurion XVI.II (Statpoint Technologies Inc., Warrenton, VA) and GraphPad Prism 7.00 (GraphPad Software, San Diego, CA). Normality was verified with the Kolmogorov-Smirnov test. Normally distributed and continuous variables were expressed as the mean ± standard error (SE). Nonnormally distributed variables were expressed as the median and interquartile range. Differences among proportions were evaluated using the *χ*^2^ test or *t*-test, as appropriate. Comparison of continuous variables between two groups was performed using Student's *t*-test (normally distributed) or Mann–Whitney test (nonnormally distributed); differences among groups were analyzed by ANOVA or Kruskal-Wallis test if nonnormally distributed.

Logistic regression analysis was used to determine the predictors of recurrence, perianal disease, surgical complications, and disease behavior. The independent variables tested include patient factors (age at diagnosis, gender, genotype, smoking habits, cigarettes/years, family history of IBD, allergies, breastfeeding, and antibiotic use in childhood), disease factors (first clinical presentation, diagnostic delay (calculated as the time between the first symptom/s due to CD and the clinical diagnosis), number of previous surgeries, presence of fistulae and/or abscesses, and disease location), pharmacological therapy factors, and other preoperative factors: presence of anaemia, appendectomy before surgery, tonsillectomy, arthritis, skin extension, neoplasia, and autoimmune disease.

All the independent variables were forced in the Cox proportional hazard model with backward elimination and applied to select the most promising subset of predictors by multiple regression.

Results of regression analysis are reported as odds ratios (ORs) with the respective 95% CI while statistical significance was set at *p* < 0.05.

## 3. Results

### 3.1. Patient's Characteristics

CARD15/NOD2 polymorphisms were found in 66 out of 191 CD patients analyzed: 53 patients were heterozygotes, carrying both a normal and a mutated sequence, and 13 individuals were found to be homozygotes.

The mean age at diagnosis was shown to be significantly different between wt (wild type) patients (33.25 ± 1.2, range 10-71) and the mutated (etero+omo) group (27.28 ± 1.28, range 11-57).

Additionally, data regarding the residence country of the patients was collected: 55.28% of wt patients live in central Italy, 41.46% in southern Italy, and 3.25% in northern Italy. The etero+omo group had a slightly higher but not significant percentage of individuals coming from central Italy (60.32%).

The distribution of never smoker, current smoker, and former smoker patients was significantly different in the two genetic groups (*p* = 0.04): compared to wt, etero+omo patients presented a higher percentage of nonsmokers (44.07% vs. 27.68%) and fewer former smokers (23.73% vs. 41.10%). Moreover, the mean smoked pack-years in smokers (number of cigarettes smoked per day/20 × number of years smoked) were 20.70 ± 3.62 for wt and 13.24 ± 2.78 in the etero+omo group (*p* = 0.0017).

About childbirth, 48 patients did not clearly remember their delivery and early-life period, and so, we consider for this analysis only the other 143 patients (7.69% reported birth by caesarean delivery, and 1.40% referred obstetrical complications); wt and etero+omo did not differ significantly. In addition, 23.78% did not receive maternal breastfeeding, with a similar frequency in the genotype groups; 34.96% have received long-lasting or repeated antibiotic treatment in childhood, more frequently in the etero+omo group (38.46% vs. 31.87%). Family IBD history documented through specialist medical genetic counselling was reported in 25.61% of the cases. Up to 20.12% declared to have one or more relatives affected by CD, 3.05% by UC, and 2.44% referred family history positive for both IBDs. The percentages of familial and sporadic cases were similar in the wt and etero+omo groups.

### 3.2. Association with Clinical Features

No differences were found between wt and etero+omo regarding the median delay (12 months for both groups), although the mean value was higher in the wt group (50.21 ± 9.23 vs. 28.67 ± 5.49 months, *p* = 0.13). However, when divided according to the type of polymorphism, an etero+omo patient presented a diagnostic delay significantly different (*p* = 0.0344); in particular, patients carrying a 3020insC polymorphism presented a larger Δ between diagnosis and surgery.

No evidence of different clinical presentation was found between the wt and etero+omo patients. The majority of them in both groups declared that the first symptom was alteration of the alvus (intestinal obstruction 29.71% or diarrhoea 18.86%); 3.43% presented with perianal fistulae, while 34.29% referred a wider spectrum of symptoms (diarrhoea, abdominal pain, weight loss, asthenia, and anaemia).

The distribution of disease location was not significantly related to the genotype: isolated ileal location was present in 57.57% of etero+omo and in 59.20% of wt patients; similar frequency in the two groups was found for colic disease (7.57 vs. 12.00%, respectively), while 34.85% of etero+omo and 28.80% of wt had ileocolic disease.

Moreover, arthritis was reported in 31.41% of the population, and skin manifestations such as erythema nodosum or pyoderma gangrenosum were present in 14.14% of the patients. The frequency of arthropathy did not show a difference between etero+omo and wt (28.69% and 32.80%, respectively, *p* = 0.32), as well as skin extension (12.12% and 15.20%, respectively, *p* = 0.33).

We documented a perianal disease in 34.03% of cases, affecting 31.20% of wt and 39.39% of etero+omo patients (Table 4). Further, 3 etero+omo and 1 wt patients referred anal fissure without fistulae; one patient reported both. The risk of perianal disease was significantly lower in patients with disease localized in the upper part of the intestine, ileum, or ileum-colon and, borderline, in those with a median of pack/year less than 6 (*p* = 0.0538) and higher in the presence of CARD15 polymorphisms (*p* = 0.0886) (Table 5). Perianal disease was correlated to both ileostomy (8.02% of our patients) and colostomy (2.14%). Smoking habits seemed to be associated with increased risk of faecal derivation: 9.43% of current smokers and 13.33% of former smokers underwent ileostomy, vs. 4.88% of never smokers (*p* = 0.12). The percentage of definitive stoma did not differ significantly between etero+omo and wt patients (12.50 and 9.68%, respectively).

Disease behavior was analyzed and correlated to genetic profile. Stricturing disease was observed in 43.98% of cases, 9.42% had fistulising behavior, and 36.13% presented both types of disease, while 10.47% had an inflammatory pattern. The etero+omo and wt groups presented similar behavior distribution (*p* = 0.75).

**Figure 2:**
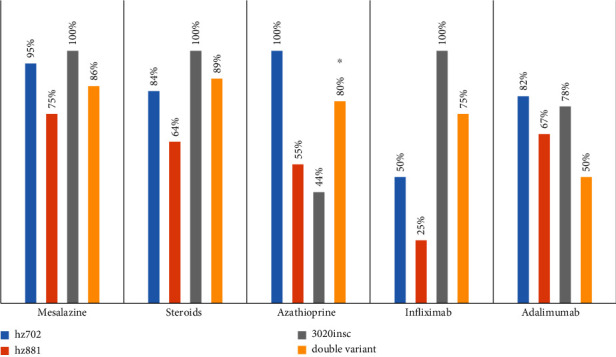
Medication responsiveness of polymorphism carriers. The figure shows the medication responsiveness of polymorphism carrier CD patients. Data are expressed as percentage. ^∗^*p* < 0.05.

Drug response was analyzed in terms of reported clinical improvement (reduction of diarrhoea and/or abdominal pain). The majority of the patient population (88.43%) had assumed mesalamine with initial good clinical results; 6.94% reported resistance to the therapy, 0.58% intolerance, and etero+omo and wt did not differ for such distribution. Regarding steroid therapy, 81.29% was responsive, 10.79% showed resistance, and 5.68% showed dependence on cortisone. No statistical difference was seen related to genotype. A similar distribution was evidenced for azathioprine, with 72.28% of responsiveness, 11.44% of resistance, and 5.42% of intolerance. In this case, we noted significant differences among etero+omo patients, according to the type of polymorphism (*p* = 0.012); those carrying an hz881 and a 3020insC allelic variant exhibited in fact a lower rate of responsiveness to azathioprine (Figure 2).

A little number of wt patients (4.71%) received methotrexate with partial benefit, while one etero+omo patient reported unresponsiveness. Similarly, 56.90% of patients assumed infliximab with good clinical results; 10.84% declared resistance, and 4.22% showed intolerance. Genotype did not correlate to particular responsiveness to infliximab. 75.41% of cases treated with adalimumab received benefit, 8.43% was resistant, and only one case resulted intolerant. History of previous emergency surgery was evaluated as a marker of disease aggressiveness: 22.73% of etero+omo and 25.60% of wt underwent at least once urgent bowel operation.

About the surgery, 92 patients (48.17%) underwent primary surgery for CD, while 99 patients (51.83%) were operated on for a surgical recurrence. Interestingly, surgical recurrence risk was significantly higher in younger patients (less than 27 years old) and those with familial IBD; on the contrary, it was significantly lower in patients with a short diagnostic delay (less than 1 year), with quiescent disease, or with no associated arthritis or surgical complications (Table 3). The presence of CARD15 variants did not change that frequency nor influenced the site of disease recurrence, which was, respectively, perianastomotic in 91.21% of cases, located elsewhere in 7.69%, and both in the site of previous resection and distally in 1.10% of recurrent patients. Regarding the number of surgical recurrence, 48.17% of patients were operated once, 27.75% twice, and 24.08% 3 or more times, with no difference related to genotype, despite the higher frequency of multiple surgeries among polymorphic patients (54.54%). Active smokers presented a higher percentage of multiple surgeries (37.78%) than never smokers (33.33%) or former smokers (28.89%). As a parameter of disease aggressiveness, for patients operated at least twice, the minimum and mean interval of time between two consecutive operations were also considered. The former presented a mean value of 5.36 years (range 0.005-23 years) without significant difference based on genotype. Similarly, the medium interval had a mean value of 6.47 years (range 0-24).

Evaluating globally primary and recurrent surgery, the type of surgical treatment in wt and etero+omo did not differ significantly: overall, 82.20% of the patients underwent resective surgery and 17.80% received resection and conservative operation (one or more strictureplasty/ies).

Furthermore, postoperative complications were reported in 30.77% of patients (48/156): anastomotic leakage occurred in 29.17% cases, anaemia-requiring blood transfusion in 14.58%, incisional hernia in 20.83%, wound infection in 12.5%, anastomotic strictures in 4.17%, and mechanical ileus in 1 case (2.08%) as short bowel syndrome, while 14.58% experienced a combination of complications.

Postoperative follow-up of the patients was performed assessing the disease status (quiescent or active inflammation) or exitus: only a wt 78-year-old woman died, because of a multiorgan failure 2 years after colic resection for Crohn colitis. At follow-up, 34.03% of patients had symptomatic active disease and 65.97% had no sign of inflammation at both clinical and instrumental follow-up (endoscopy and MRI). No statistical significance resulted from the comparison of the outcome in the etero+omo and wt groups.

History of malignancies was observed in 7.3% of patients, with no significant difference by genetic profile: cancer of the gastrointestinal tract was observed in five patients, and 9 extraintestinal sites of tumours were observed. Among wt patients, one woman presented a rectal adenocarcinoma associated with hereditary nonpolyposis colorectal cancer syndrome, 1 woman was operated on for breast cancer at the age of 20, a heavy smoker man had larynx cancer, two men were operated on for prostate tumour, two men had papillary kidney carcinoma, and one man had papillary thyroid carcinoma. Among etero+omo patients, a male patient heterozygote for 3020insC polymorphism underwent surgery for colloid colon carcinoma which developed on an ulcerated stenosis after 24 years of Crohn colitis. Breast cancer occurred at a young age for a woman with heterozygotes for 3020insC. A never smoker man carrier of G881R heterozygosis and affected by ileocolic CD since the age of 29 had bladder cancer at the age of 53. A woman hzR702W suffering from colic CD since the age of 36, when she underwent vulvectomy for a rare cancer of the clitoris, and at the age of 68 was operated for rectal adenocarcinoma ex tubule-villous adenoma.

The presence of autoimmune disease (Hashimoto thyroiditis, psoriasis, and vitiligo) showed identical frequency in both etero+omo and wt groups, 13.6%. Allergies were reported in 25.65% of the study population, with a higher but not significantly different frequency among etero+omo patients (31.82 vs. 22.4% of wt). Even asthma, occurring in 4.19% of the cases, showed no association with genotype.

History of appendectomy performed at a young age was recorded and correlated to genotype. In the etero+omo group, 33.93% of cases underwent surgery for acute appendicitis, as well as 24.52% of wt (*p* = 0.20). Moreover, history of tonsillectomy was investigated as a condition indicating a possible childhood immune alteration associated with oral microbiota modification. Etero+omo patients declared to have undergone tonsillectomy in 22.64% of cases, which is fewer than wt (32.65%). This finding, though not significant (*p* = 0.19), suggest a potential contribution of the removal of tonsils in the patient immunological alterations.

## 4. Discussion

IBD-associated loci account for only 13.60% of disease variance in CD, the rest resulting from the environment or “hidden heritability” caused by genetic interactions or epigenetics [5, 10, 49]. In our series, polymorphisms of CARD15/NOD2 were detected in 66 patients (34.55% of the study population). We did not find a phenotypic difference between CD patients who were either homozygous for one or compound heterozygous for any of the R702W, G908R, and L1007fs variants, suggesting that the respective roles of these variants are comparable.

According to a systematic review performed by Loftus et al. among North American IBD patients, the mean age for CD diagnosis ranged from 33 to 45 years [50]. Moreover, a recent study investigating all known 163 IBD risk gene loci has found that increased genetic burden was associated with early CD diagnosis [51].

In our cohort, the mean age at the time of diagnosis was 31.09 years, but mutant CARD15 subjects were significantly younger compared to wild type patients: 27.28 years versus 33.25, respectively (*p* = 0.004). As previously reported, the risk for CD is 13-fold higher in first-degree relatives of individuals affected by CD [52]. In our cohort, familial history of IBD was observed in 25.61% of cases, with similar frequency in the two genotype groups, and remarkable phenotypic concordance among affected relatives was reported, except for 3.05% of cases who referred familial history positive for UC. In agreement with published studies, we observed that familial IBD history increased recurrence risk (OR = 2.14; *p* = 0.016) together with a younger age at diagnosis (OR = 3; *p* = 0.0086).

Several authors reported a less frequent involvement of the colon in patients carrying CARD15 polymorphisms, and ileal location was predicted by higher genetic burden in CD [53, 54]. Accordingly, ileal CD is allegedly determined in part by genetic factors, while CD with colonic localization could be explained more by environmental factors [55]. However, in our series, the genotype is not associated with a particular disease location.

Ileal CD patients have been demonstrated to present reduced levels of the antimicrobial peptide alpha-defensin originating from Paneth-cells [56]. This decrease was more evident in patients with a 1007 fs variant, suggesting an altered intestinal microbiota and dysregulated innate immunity. Studies conducted on a murine model suggest that NOD2 polymorphisms may predispose to CD by decreasing tolerance to the resident microbial flora [57]. Indeed, CD in patients carrying at least one variant of CARD15 tends to show a more aggressive clinical course [58]. In our study, a retrospective assessment of a possible early-life dysbiosis was obtained through a multi-item questionnaire investigating common childhood conditions associated with microbial impairment, such as caesarean delivery, artificial breastfeeding, and long-term use of antibiotics [59]. In our cohort, several patients claimed one or more of these factors, independently of the genotype, in particular one-third of them, the antibiotic use in childhood, thus suggesting the dysbiosis as a pathogenic factor. However, the evidence supporting a causative role of *in utero* or perinatal measles in CD development is still insufficient. Our enrolled patients are Caucasian subjects living in urban areas, mostly in central and southern Italy, with medium to high social status and hygiene conditions. A recent study has identified higher social status, higher educational level of parents, and living in urban areas as important risk factors for CD and complications [60, 61].

We included surgical patients, which are complicated CD cases refractory to medical treatments or with severe organic complications (fistula-abscess, severe stricture) characterized according to the parameters indicated by the Montreal classification. In this cohort, we further analyzed the presence of possible risk factors that could influence the postoperative course, promoting recurrence.

An extensive survey investigating genetic and clinical factors predisposing to perianal manifestations of CD identified colic CD location as the only significant predictor of perianal disease [62]. In agreement, our data showed that perianal disease risk was lower in patients with ileo and ileocolon CD location (*p* < 0.001). Genetic variations within the CARD15/NOD2 resulted in a higher but not significant risk factor for perianal CD (OR = 1.87; *p* = 0.0886). Studies on perianal disease in CD contrast regarding gender as a risk factor for perianal fistulae. In our cohort, the female/male ratio in patients affected by perianal disease was 0.80 and not significantly different from the gender ratio of the whole cohort (0.74). On the contrary, we noted a borderline significant association between perianal disease risk and the smoke habit (*p* = 0.0538), suggesting an increased risk for heavy smokers.

Evaluating our results, we observed several clinical factors associated with a more aggressive natural CD history, such as (i) the development of early-onset disease leading to complications requiring surgery a few years after diagnosis, (ii) the presence of extraintestinal disease and perianal fistula, and (iii) the necessity of a permanent stoma. The latter are factors related to the development of single or multiple surgical recurrences during follow-up. Even if we did not find significant association between CARD15 polymorphisms and disease location, disease behavior, perianal fistulae, risk of permanent stoma, number of reoperations, and extraintestinal manifestations, analyzing the diagnostic delay of etero+omo patients, according to the type of polymorphism, we found that patients carrying a 3020insC polymorphism presented a larger Δ between diagnosis and surgery (*p* = 0.0344). This data indicate that their CD course could be characterized by a lower rate of complications that usually bring patients to surgery. On the contrary, the study of Bhullar et al. performed in a cohort of only 30 CD patients reported that patients with 3020insC polymorphism had a significantly reduced time between their diagnosis and first surgical resection, suggesting that the polymorphism leads to a more aggressive disease form, which rapidly progresses requiring early surgical intervention [13]. On the other hand, another study reported that children with the 3020insC polymorphism have a 6.6-fold increased risk for developing a stricturing phenotype requiring surgery [63].

Interestingly, we also noted, for the first time, that patients carrying an hz881 and a 3020insC exhibited a lower rate of responsiveness to azathioprine (*p* = 0.012), but no difference was found in biologic therapy (Figure 2). About azathioprine, we observed a decrease in responsiveness of 20%, statistically significant in double polymorphism carriers (Table 3). This evidence is clinically relevant as azathioprine is commonly used in CD patients. Therefore, a preventive screening of the double polymorphism carriers could avoid both delays in starting postoperative therapy with biological drugs and the development of adverse events due to azathioprine administration, thus maximizing the cost-effectiveness of medical therapy and tailoring the efficacy on the patients' characteristics.

Currently, many studies have reduced the importance of the genetic component in the pathogenesis of Crohn's disease, but this study underlines the importance of considering not only the single mutation but also the associations between them in the various susceptibility genes known.

The detection of particular combinations of variants related to the clinical course or treatment responsiveness could allow the design of new genetic biomarkers, prognostic or theranostic, leading to a more personalized approach in CD.

In conclusion, the present study confirms that in Caucasian patients, CD natural history seems not strictly related to genetic factors; however, CARD15 polymorphisms are associated with an earlier CD onset, and both age at diagnosis < 27 years and familiarity for CD were confirmed to have a detrimental effect on the postoperative natural history of the patients. Differences in relation to the three polymorphisms have been also detected in our cohort, but essentially, we documented for the first time that patients carrying an hzG881R and a 3020insC exhibited a lower rate of responsiveness to azathioprine with relevant clinical-correlated impact.

## Figures and Tables

**Table 1:** Demographic and clinical features of CD patients.

	All cases	Wild type (WT)	Polymorphism carriers (etero+omo)
Number of patients	191	125	66
Age of onset (yrs)	27 (21.5-38.5)	28 (23-43.5)	25 (19.25-34)^∗^
Δ diagnosis-surgery (months)	12 (6-36)	12 (5-40)	12 (6-36)
Gender			
Male	110 (58%)	68 (54%)	42 (64%)
Female	81 (42%)	57 (46%)	24 (36%)
Smoking habit			
No	57 (33%)	31 (28%)	26 (44%)^∗^
Yes	54 (32%)	35 (31%)	19 (32%)
Former	60 (35%)	46 (41%)	14 (24%)
Pack years	15 (6-27)	18 (10-35)	9 (5-18)^∗∗^
Disease location			
Ileo	112 (59%)	74 (59%)	38 (57%)
Colon	20 (10%)	15 (12%)	5 (8%)
Ileocolon	59 (31%)	36 (29%)	23 (35%)
Disease behavior			
Inflammatory	20 (11%)	13 (10%)	7 (11%)
Stricturing	84 (44%)	57 (46%)	27 (41%)
Fistulising	18 (9%)	10 (8%)	8 (12%)
Stricturing and fistulising	69 (36%)	45 (36%)	24 (36%)
Extraintestinal disease			
Skin	27 (14%)	19 (15%)	8 (12%)
Arthritis	60 (31%)	41 (33%)	19 (29%)
Perianal fistulae	65 (34%)	39 (31%)	26 (39%)
Medication responsiveness			
Mesalazine	140 (92%)	94 (93%)	46 (88%)
Steroids	126 (81%)	80 (81%)	46 (82%)
Azathioprine	73 (72%)	44 (72%)	29 (73%)
Infliximab	33 (57%)	20 (54%)	13 (62%)
Adalimumab	46 (75%)	24 (75%)	22 (76%)
Type of surgery			
Resection	157 (82%)	100 (80%)	55 (83%)
Resection+SXPL	34 (18%)	25 (20%)	11 (17%)
Recurrence	99 (52%)	64 (51%)	35 (53%)
Multiple operations (≥2)	99 (52%)	63 (50%)	36 (55%)
History of urgent surgery	47 (25%)	32 (26%)	15 (23%)
Minimum surgery-free interval (yrs)	3 (0.5-9)	3 (0.5-8)	3 (0.2-13.5)
Mean surgery-free interval (yrs)	5 (2-9)	5 (2-8)	5 (2-13.5)
Digestive cancer	5 (3%)	3 (2%)	2 (3%)
Extraintestinal cancer	9 (5%)	7 (6%)	2 (3%)
Immunological comorbidity			
Autoimmunity	26 (14%)	17 (14%)	9 (14%)
Asthma	8 (4%)	4 (3%)	4 (6%)
Allergies	49 (26%)	28 (22%)	21 (32%)
Familial IBD	42 (26%)	29 (27%)	13 (24%)
Disease status			
Quiescent	95 (66%)	56 (62%)	39 (74%)
Active	49 (34%)	35 (38%)	14 (26%)

Data are median (interquartile range 25–75) for continuous variables and number (percentage) for categorical variable. ^∗^*p* < 0.05, ^∗∗^*p* < 0.01 WT vs. etero+omo, by *χ*^2^ or Mann–Whitney test.

**Table 2:** Primers and PCR condition used for the detection of R702W, G881R, and 3020insC polymorphisms in NOD2 gene.

Exon	Polymorphism	Primers	Annealing temperature
4	Arg702Trp (R702W)	5′-AGATCACAGCAGGCCTTCCTG-3′ 5′-GCCAATGTCACCCACAGAGT-3′	59°C
8	Gly881Arg (G881R)	5′-AAGTCTGTAATGTAAAGCCAC-3′ 5′-CCCAGCTCCTCCCTCTTC-3′	55°C
11	3020insC	5′-CTCACCATTGTATCTTCTTTTC-3′ 5′-GAATGTCAGAATCAGAAGGG-3′	55°C

**Table 3:** Demographic and clinical features of polymorphism carriers.

Type of polymorphism	R702W	G881R	3020insC	Double variant
Number of patients	25	15	13	10
Age of onset (yrs)	24.5 (19-31.25)	29 (20-38)	22.5 (19.25-26.5)	28 (16-42.5)
Δ diagnosis-surgery (months)	12 (4-12)	10 (6-43)	36 (12-84)	24 (12-55)^∗^
Gender				
Male	15 (60%)	10 (67%)	6 (46%)	9 (90%)
Female	10 (40%)	5 (33%)	7 (54%)	1 (10%)
Smoking habit				
No	6 (27%)	10 (72%)	7 (58%)	3 (33%)
Yes	9 (41%)	2 (14%)	3 (25%)	4 (45%)
Former	7 (32%)	2 (14%)	2 (17%)	2 (22%)
Disease status				
Quiescent	17 (85%)	8 (61%)	9 (82%)	3 (43%)
Active	3 (15%)	5 (39%)	2 (18%)	4 (57%)
Disease location				*p* = 0.0527
Ileo	15 (60%)	8 (53%)	11 (84%)	2 (20%)
Colon	2 (8%)	0	1 (8%)	2 (20%)
Ileocolon	8 (32%)	7 (47%)	1 (8%)	6 (60%)
Disease behavior				
Inflammatory	1 (4%)	1 (7%)	1 (8%)	1 (12%)
Stricturing	11 (46%)	7 (46%)	3 (25%)	4 (44%)
Fistulising	2 (8%)	1 (7%)	3 (25%)	2 (22%)
Stricturing and fistulising	10 (42%)	6 (40%)	5 (42%)	2 (22%)
Extraintestinal disease				
Skin	4 (19%)	2 (14%)	1 (9%)	1 (14%)
Arthritis	7 (33%)	4 (28%)	5 (45%)	2 (28%)
Perianal fistulae	10 (40%)	4 (27%)	4 (33%)	6 (60%)
Medication responsiveness				
Mesalazine	18 (95%)	9 (75%)	11 (100%)	6 (86%)
Steroids	16 (84%)	9 (64%)	11 (100%)	8 (89%)
Azathioprine	15 (100%)	5 (55%)	4 (44%)	4 (80%)^∗^
Infliximab	4 (50%)	1 (25%)	5 (100%)	3 (75%)
Adalimumab	9 (82%)	4 (67%)	7 (78%)	1 (50%)
Type of surgery				
Resection	18 (86%)	12 (80%)	10 (83%)	9 (90%)
Resection+SXPL	3 (14%)	3 (20%)	2 (17%)	1 (10%)
Recurrence	9 (43%)	8 (53%)	8 (67%)	6 (67%)
Multiple operations (≥2)	9 (41%)	9 (60%)	8 (67%)	8 (80%)
History of urgent surgery	2 (9%)	5 (33%)	3 (25%)	*p* = 0.673 5 (50%)
Minimum surgery-free interval (yrs)	5.058 ± 2.02	7.796 ± 2.8	7.886 ± 2.6	4.157 ± 2.7
Mean surgery-free interval (yrs)	5.81 ± 1.9	8.133 ± 2.7	9.438 ± 2.2	5.957 ± 2.5
Digestive and/or extraintestinal cancer	1 (4%)	1 (7%)	2 (14%)	0
Immunological comorbidity				
Autoimmunity	3 (15%)	1 (8%)	3 (27%)	1 (14%)
Asthma	1 (5%)	0	2 (18%)	1 (14%)
Allergies	9 (45%)	5 (36%)	6 (54%)	1 (12%)
Familial IBD	6 (33%)	5 (36%)	1 (10%)	1 (11%)

Data are mean ± SE or median (interquartile range 25–75) for continuous variables and number (percentage) for categorical variable. ^∗^*p* < 0.05, ^∗∗^*p* < 0.01 WT vs. etero+omo, by *χ*^2^ or Kruskal-Wallis test.

**Table 4:** Independent variables associated to the perianal disease risk.

Perianal disease risk	Odds ratio	95.0% confidence intervals		*p* value
		Lower limit	Upper limit	
Site				0.0007
Ileum	0.15	-2.94017	-0.802703	
Ileocolon	0.36	-2.12092	0.0871799	
Pack/years < 6 (yrs)	0.50	-1.38701	0.0248437	0.0538
Genotype	1.87	-0.103337	1.36101	0.0886

**Table 5:** Independent variables associated with the surgical recurrence risk.

Recurrence risk	Odds ratio	95.0% confidence intervals		*p* value
		Lower limit	Upper limit	
Age at diagnosis < 27 yrs	3.00	0.251699	1.94821	0.0086
Diagnostic delay < 12 months	0.35	-1.93322	-0.180071	0.0139
No arthritis	0.394	-1.77804	-0.0821999	0.0276
No surgical complications	0.292	-2.14659	-0.314302	0.0062
Follow-up (quiescent disease)	0.231	-2.35501	-0.576888	0.0007
Familial IBD	2.14	0.171336	2.25389	0.0160

## Data Availability

The underlying data supporting our study will be shown after a via email request.
